# Supplementary material for: A large language model for clinical outcome adjudication from telephone follow-up interviews: a secondary analysis of a multicenter randomized clinical trial
Source: Nat Commun. 2025 Dec 1;17:211. doi: 10.1038/s41467-025-66910-6 (PMC12779956; doi:10.1038/s41467-025-66910-6)
Supplement: Supplementary file 1 — Supplementary Information [file 41467_2025_66910_MOESM1_ESM.pdf]

Title: A Large Language Model for Clinical Outcome Adjudication from Telephone Follow-up Interviews: A Secondary Analysis of a Multicenter Randomized Clinical Trial

Supplementary Materials

Supplementary Methods

Supplementary Method 1. Consensus for establishing the silver reference standards

In China CT-FFR Study 3, the primary endpoint was the invasive coronary angiography (ICA) rate at 90 days [1]. The secondary endpoint was major adverse cardiovascular events (MACE) during 90 days, 1-year cardiac events and 1-year revascularization. Therefore, a joint meeting of experts from the core laboratory and Clinical Events Committee (CEC) of CHINA CT-FFR 3 trial determined the following event that should be adjudicated: death event; hospitalization event, from which the admission note or discharge summary can be further collected and analyzed, and only for hospitalization due to cardiovascular diseases; surgery event, from which ICA event could be traced and the admission note or discharge summary can be further collected; and medication use. In order to evaluate the reliability of the telephone follow-up, whether the participant himself / herself who responded to the phone and answered the follow-up questions was also adjudicated. A consensus was developed by the core laboratory and CEC for establishing the adjudications only according to telephone interview recordings as following:

(1) **Information source** (whether the information came from the participant themselves): If it was the participant himself / herself who responded to the phone and answered the follow-up questions, the judgment is "Yes". If a relative to the participant responded to the phone and the follow-up questions, it would be judged as "No".

(2) **Death event** (whether the participant died during the follow-up period): If the relative to the participant explicitly mentioned the participant’s death, it was considered "Yes", otherwise it was considered "No". In the death cases, for humanitarianism reason, the follow-up staff would not inquire about the information of hospitalization, surgery or medication during telephone interview, so these three events for the death cases would not be evaluated. And

the medical records (admission note or discharge summary, if any) for all deaths would be traced by the site investigators.

(3) **Hospitalization event** (whether the participant was hospitalized during the follow-up period): A clear mention of hospitalization would be considered "Yes", while a denial of hospitalization be considered "No". For definite operations such as intracoronary stent implantation, coronary artery bypass grafting, ICA, both hospitalization and surgical events can be inferred and marked as "Yes". The only mention of “angiography” without specifying whether it is digital subtraction angiography (DSA) or CT angiography (CTA), as well as the health examination and community hospital examination, cannot be inferred as hospitalization. If there was no follow-up examination at all, hospitalization ought to be adjudicated as "No". Without any relevant information mentioned, it should be judged as "Not mentioned". Those cases that cannot be evaluated based on the dialogue because of logical confusion or unclear expression of the participants would be judged as "Uncertain". And the medical records (admission note or discharge summary, if any) for hospitalization due to cardiovascular diseases would be traced by the site investigators.

(4) **Surgical event** (whether the participant underwent surgery during the follow-up period): A definite mention of surgery, such as intracoronary stent implantation, coronary artery bypass grafting, invasive cerebral angiography and invasive coronary angiography, can be judged as "Yes". While a denial of surgery be considered "No". When referring to prescription only or optimal medical therapy only, we tend to consider hospitalization as "No". Without any relevant information mentioned, the judgement would be "Not mentioned". If the participant’s logic was chaotic or contradictory, making it impossible to make a definite judgement, it would be considered "Uncertain". And the medical records (admission note or discharge summary, if any) for all surgical events would be traced by the site investigators.

(5) **Medication event** (whether the participant took medication during the follow-up period): During the follow-up period, the medication event would be considered "Yes" as long as the participant has taken medication regardless of whether the drug has been discontinued or not. Without any relevant information mentioned, the judgement was "Not mentioned". If the participant’s logic is chaotic or contradictory, making it impossible to make a judgement, it is considered "Uncertain".

Supplementary Method 2. Definitions for primary and secondary outcomes of China CT-FFR Study 3

In China CT-FFR Study 3, follow-up was conducted by the follow-up staffs. All follow-up was performed at 90 days and 1 year after enrollment at the study sites by means of telephone interview or electronic medical records until February 28, 2022 and November 30, 2022. The clinical and outcomes data were collected by personnel at the core laboratory at Jinling Hospital, and an independent clinical events committee adjudicated all primary and secondary endpoint events in a blinded fashion. The independent clinical events committee was blinded to randomized group information, CCTA images, and the CT-FFR report but not to other information (eg, clinical history and results of electrocardiographic and laboratory examinations). The clinical events committee (including four clinicians with 12-30 years of experience and one radiologist with 20 years of experience) adjudicated all primary and secondary end points based on standardized, prospectively determined definitions [1].

The primary endpoint of the study was rates of ICA regardless of coronary revascularization within 90 days after CCTA.

The secondary endpoints included (i) a composite of MACE composed of all-cause mortality, non-fatal myocardial infarction (MI), and urgent revascularization, and (ii) a composite of 1-year cardiac events including cardiac death, non-fatal acute MI and urgent revascularization. (iii) 90-day and 1-year revascularization.

All-cause mortality: All-cause death is defined as death from any cause in a certain period of time [2].

Non-fatal myocardial infarction (MI): The third universal definition of myocardial infarction (MI) of the ESC/ACCF/AHA/WHF Task Force was implemented [3]. An event is defined as nonfatal if it does not cause the affected participant's death. All fatal events will be recorded as all-cause death. The preferred biomarker for the MI definition is cardiac troponin I or T (cTn). If a cTn assay is not available, the best alternative is creatine kinase MB isoform (CK_{MB}).

Urgent revascularization: Urgent revascularization is any subsequent revascularization following the initial treatment decision at index ICA and occurring at a minimum of 6 weeks after index ICA [4].

Supplementary Method 3. Three prompts to output the preadjudications

3.1 zero_shot_prompt

Parse follow-up reports based on follow-up dialogue and provide structured results.

# Fields to Extract

1. Source of follow-up information
2. Whether the participant is deceased
3. Whether the participant was hospitalized
4. Whether the participant underwent surgery
5. Whether the participant took medication

# Field Definitions and Determination Methods

1. Source of follow-up information

- **Definition:** Whether the follow-up participant is the person themselves
- **Value Range:** ["Self", "Relative"]
- **Determination Method:** Comprehensively analyze the dialogue content to determine if the follow-up was received by the participant themselves.

2. Whether the participant is deceased

- **Definition:** Life status of the participant
- **Value Range:** ["Yes", "No"]
- **Determination Method:**
    - "Yes": Relative explicitly mentions the participant's death.
    - "No": All other situations.

3. Whether the participant was hospitalized

- **Definition:** Whether the participant received inpatient treatment during the follow-up period
- **Value Range:** ["Yes", "No", "Not Mentioned", "Uncertain"]
- **Determination Method:**

- "Yes":
  - Explicit mention of hospitalization.
  - Situations implying hospitalization: Stent placement, bypass surgery, valve replacement, invasive cerebral angiography.
- "No":
  - Explicit denial of hospitalization.
  - Mention of no follow-up exams or only health check-ups.
  - Mention of angiography without specifying DSA or CTA.
  - Treatment received only at a community hospital.
- "Not Mentioned": No relevant information.
- "Uncertain": Participant’s illogical or contradictory statements preventing a clear judgment.

4. Whether the participant underwent surgery

- **Definition:** Whether the participant received surgical treatment during the follow-up period
- **Value Range:** ["Yes", "No", "Not Mentioned", "Uncertain"]
- **Determination Method:**
  - "Yes":
    - Explicit mention of surgery.
    - Specific surgery types: stent, bypass, valve replacement, invasive cerebral angiography, etc.
  - "No":
    - Explicit denial of surgery.
    - Mention of only medication prescription or conservative treatment.
  - "Not Mentioned": No relevant information.
  - "Uncertain": Participant’s illogical or contradictory statements preventing a clear judgment.

5. Whether the participant took medication

- **Definition:** Whether the participant used medication during the follow-up period
- **Value Range:** ["Yes", "No", "Not Mentioned", "Uncertain"]
- **Determination Method:**
    - "Yes": Medication used during follow-up (regardless of discontinuation).
    - "No": All other situations.
    - "Not Mentioned": No relevant information.
    - "Uncertain": Participant's illogical or contradictory statements preventing a clear judgment.

# Output Structure

Structured results in JSON format, ensuring it is a directly parseable dictionary (Dict[str, str]):

```json
{{
"Source of follow-up information":"XX",
"Whether the participant is deceased":"XX",
"Whether the participant was hospitalized":"XX",
"Whether the participant underwent surgery":"XX",
"Whether the participant took medication":"XX"
}}
```

# Important Notes

1. The four events (death, hospitalization, surgery, medication) must be judged independently; conclusions cannot be inferred from one another.
2. While surgery and hospitalization events are judged independently, certain specific surgeries may simultaneously imply hospitalization.
3. Special case: If "Whether the participant is deceased" is "Yes", only retain these two fields: {"Source of follow-up information": "XX", "Whether the participant is deceased": "XX"}. Do not include extra fields.
4. Strictly follow the specified output structure.

3.2 zero_shot_cot_prompt

Based on the follow-up conversation, parse the follow-up report according to the reasoning process. Finally, provide the reasoning thought process and structured results.

# Fields to Extract

1. Source of follow-up information
2. Whether the participant is deceased
3. Whether the participant was hospitalized
4. Whether the participant underwent surgery
5. Whether the participant took medication

# Field Definitions and Determination Methods

(Identical to Document 4.1 zero_shot_prompt above)

# Output Structure

The output should include 2 parts:

Part 1: Under the title ## Reasoning, describe the reasoning process in detail.

Part 2: Structured results output in JSON format, ensuring it is a directly parseable dictionary (Dict[str, str]), formatted as follows:

```json
{{
"Source of follow-up information":"XX",
"Whether the participant is deceased":"XX",
"Whether the participant was hospitalized":"XX",
"Whether the participant underwent surgery":"XX",
"Whether the participant took medication":"XX"
}}
```

Important Notes

1. The four events (death, hospitalization, surgery, medication) must be judged independently; they cannot be inferred from each other.
2. Surgery and hospitalization events are judged independently, but specific surgeries can simultaneously imply hospitalization.
3. Specifically, if the prediction for 'Whether the participant is deceased:' is "Yes", then only retain the following two pieces of information: {"Source of follow-up information":"XX", "Whether the participant is deceased":"XX"}. Do not include extra information.
4. Think according to the reasoning process below, then output the result according to the output structure

Reasoning Process

Refer to the following reasoning process to derive the answers:

When answering each question, think step by step. First, locate key statements useful for judgment, then sequentially compare against the determination methods in # Field Definitions and Determination Methods. Select the most appropriate option from the value range as the answer, and output according to the output structure.

3.3 one_shot_prompt

Based on the follow-up conversation, parse the follow-up report by referencing the example. Finally, provide structured results.

Fields to Extract

1. Source of follow-up information
2. Whether the participant is deceased
3. Whether the participant was hospitalized
4. Whether the participant underwent surgery
5. Whether the participant took medication

Field Definitions and Determination Methods

(Identical to Document 4.1 zero_shot_prompt above)

Output Structure

Structured results output in JSON format, ensuring it is a directly parsable dictionary (Dict[str, str]), formatted as follows:

```json
{{
"Source of follow-up information":"XX",
"Whether the participant is deceased":"XX",
"Whether the participant was hospitalized":"XX",
"Whether the participant underwent surgery":"XX",
"Whether the participant took medication":"XX"
}}
```

Important Notes

(Identical to Document 4.1 zero_shot_prompt above)

Important Notes

1. The four events (death, hospitalization, surgery, medication) must be judged independently; they cannot be inferred from each other.
2. Surgery and hospitalization events are judged independently, but specific surgeries can simultaneously imply hospitalization.
3. Specifically, if the prediction for 'Whether the subject is deceased:' is "Yes", then only retain the following two pieces of information: {"Source of follow-up information":"XX", "Whether the participant is deceased":"XX"}. Do not include extra information.
4. Follow the example below and output the result according to the output structure requirements.

Example

Input Follow-up Dialogue:

"Role1: Hello.\nRole2: Hello, may I ask who you are? Hello, may I ask if you are ###### (phonetic)?\nRole1: Hello, who is this?\nRole2: This is Nanjing First Hospital. Didn't you have a coronary artery examination here on June 5th last year? I'm conducting a follow-up on your recent condition.\nRole1: Oh.\nRole2: How have you been feeling lately?\nRole1: I said I haven't had any checkups since then.\nRole2: None, right? How have you been feeling recently? Any chest pain, chest tightness, or discomfort?\nRole1: I've been taking medication.\nRole2: After taking the medication, any chest pain, chest tightness, or discomfort?\nRole1: No.\nRole2: Everything's been fine, right?\nRole1: Yes.\nRole2: No follow-up checks from last year until now, right?\nRole1: Yes.\nRole2: Have you had any physical exams?\nRole1: No.\nRole2: No follow-ups, no physical exams, and feeling pretty good after taking the medication?\nRole1: Yes, that's right.\nRole2: Haven't had any surgeries either, right?\nRole1: No.\nRole2: What medications are you taking now?\nRole1: I take that one, what's it called?\nRole2: Statin.\nRole1: Mm-hmm.\nRole2: So just one statin?\nRole1: Yes, just the one (00:01:20).\nRole2: Just one statin, right?\nRole1: Yes.\nRole2: And your condition has been good recently, no discomfort?\nRole1: Yes.\nRole2: Okay, good. Thank you for your cooperation.\n00:01:36\nEnd"

Output Result:

```json
{{
"Source of follow-up information":"Self",
"Whether the participant is deceased":"No",
"Whether the participant was hospitalized":"No",
"Whether the participant underwent surgery":"No",
"Whether the participant took medication":"Yes"
}}
```

Supplementary Method 4. Example of Several Patterns for the Samples that Fu-LLM Gets Wrong

1. The dialogue is too shot

Input Follow-up Dialogue:

"Role1: Hello, is this Mr./Ms. ### (phonetic)?

Role2: No, may I ask why?

Role1: Are you a family member?

Role2: I'm her maternal aunt.

Role1: All right. Regarding Ms. ### from your family – she underwent a coronary CTA examination at our hospital in 2021. Do you recall?

Role2: She hasn't done the follow-up check yet. She mentioned going but didn't.

Role1: I understand she completed the initial exam last year. I'm calling to follow up: Has she experienced chest pain since the examination?

Role2: I don't know. (00:01:05) Let me check with her children first, all right?

Role1: Very well, I'll contact others instead.

Role2: Okay."

Interpretation: According to the text, the silver reference for the question of "Whether the participant was hospitalized" was Not-mentioned. While Fu-LLM response No. This could contribute to a very short conversation.

2. Confusion of the chronological order of events

Input Follow-up Dialogue:

"Role1: Hello, is this Mr./Ms. ### (phonetic)?

Role2: Yes.

Role1: Hello, I'm calling from Jining Hospital.

Role2: Mm-hmm.

Role1: To follow up: You underwent a coronary CT scan last year for cardiac evaluation. Could I briefly inquire about your recent health status?

Role2: No recent health issues.

Role1: All good then?

Role2: Mm-hmm.

Role1: Any chest pain episodes this past year?

Role2: None.

Role1: Cardiac health stable overall?

Role2: Heart's fine, just gastric issues.

Role1: Have you had any follow-up examinations since?

Role2: No.

Role1: Confirming no? Any surgical procedures?

Role2: No surgeries, only hysterectomy.

Role1: Hysterectomy performed?

Role2: Mm-hmm.

Role1: How many years ago?

Role2: Over 40 years.

Role1: About 40 years?

Role2: Over forty years actually.

Role1: Over forty years? Any current medications?

Role2: I have hypertension.

Role1: Antihypertensive medication then?

Role2: Mm-hmm.

Role1: Remember the drug name?

Role2: Taking... (00:01:28)

Role1: (00:01:28) Understood?

Role2: Mm-hmm.

Role1: Brief follow-up complete. Wishing you good health. Goodbye.

Role2: Goodbye."

Interpretation: According to the text, the silver reference for the question of "Whether the subject was hospitalized" was No. While Fu-LLM response Yes. This could contribute to that the participant had hysterectomy forty years ago, while the Fu-LLM could not separate it from

hospitalization during the follow-up.

3. AI Hallucinations

Input Follow-up Dialogue:

"Role1: Hello, is this ### (phonetic)?

Role2: Who are you from?

Role1: I'm calling from Jining Medical University Affiliated Hospital, conducting a brief telephone follow-up to inquire about your recent health status. Are you the participant?

Role2: He's back in our hometown, not here anymore.

Role1: So you're currently not living together, correct?

Role2: That's right.

Role1: Are you aware of his current health condition?

Role2: He's doing alright now.

Role1: Has he experienced any episodes of chest pain?

Role2: I don't think so.

Role1: None, correct? Has he undergone any follow-up examinations recently?

Role2: No. You know… the pandemic.

Role1: Right, none during the pandemic. So no checkups in the past six months?

Role2: Probably not.

Role1: Confirming none? No cardiac-related examinations?

Role2: No.

Role1: Nor any surgical procedures, correct?

Role2: Anything else to add? I might forget later.

Role1: Understood."

Interpretation: According to the text, the silver reference for the question of "Whether the participant is deceased" was No. While Fu-LLM response Yes. This could contribute to AI Hallucinations.

4. Mistakes occurred in voice-to-text conversions

Input Follow-up Dialogue:

"Role1: Hello, is this the family of ### (phonetic)? Hello, I'm calling from Jining Medical University Affiliated Hospital. He had a coronary CT scan last year - a heart examination. We're following up to ask about his recent health status.

Role2: He's been taking medication, but sometimes experiences palpitations.

Role1: Palpitations? Has he returned to the hospital for further examination?

Role2: (00:01:01)

Role1: He should continue following the treatment plan from his specialist doctor - including when to schedule follow-ups and which medications to take. Our call is primarily to check on his recent health status.

Role2: (00:01:15 dialect)

Role1: Has he undergone any surgical procedures?

Role2: No, no surgeries.

Role1: What medication is he currently taking?

Role2: (00:01:25 dialect)

Role1: Could you clarify what medication he's taking?

Role2: (00:01:28 dialect)

Role1: Alright, thank you. We'll continue monitoring his health. Goodbye.

(End of call at 00:01:37)"

Interpretation: There was some mistakes occurred in voice-to-text conversions, and some information could not be recognized. According to the text, the silver reference for the question of “Whether the participant was hospitalized” was Not-mentioned. While Fu-LLM response No. This could contribute to the unrecognized information in voice-to-text conversions.

Supplementary Method 5. Prompt Used for Data Rewrite

Task

Based on the Follow-up Report Results and the Judgment Criteria, retrospectively analyze the Follow-up Dialogue Content. Optimize the dialogue by ensuring all key Q&A segments, which are necessary to directly or indirectly determine the results in the report, are preserved. Concurrently, randomly delete half of the non-key Q&A segments to reduce data volume without sacrificing important information. Only return the content of the processed follow-up dialogue, without any other redundant explanations or descriptions.

Follow-up Report Results

{output_text}

Judgment Criteria

{common_prefix}

Follow-up Dialogue Content

{input_text}

The prompt for data re-write. Where {output_text} represents the original structured results of the follow-up report; {common_prefix} represents the judgment criteria defined in **Supplementary method 3**; and {input_text} represents the original, full content of the follow-up dialogue.

Supplementary Method 6. Prompt Used for Data Synthesis

Task Requirements

Please generate a new and unique follow-up dialogue based on the provided guidelines and content.

Follow-up Dialogue Content Guidelines

1. Relevance

The new dialogue must be closely aligned with the Expected Follow-up Report Results. The primary constraint is that analyzing the generated dialogue using the Judgment Criteria must yield results that are identical to the Expected Follow-up Report Results.

2. Dialogue Content and Style Constraints

1. Roles: A hospital staff member (typically from the Radiology or Follow-up Department) and a Patient/Family Member, marked as Role 1 and Role 2. The two roles are not familiar with each other.

2. Scenario: A telephone-based follow-up call taking place in China.

3. Purpose: To understand the patient's health status, follow-up examinations, medication usage, and symptoms.

4. Content:

    * The conversation must revolve around post-Coronary CTA check-ups, heart-related conditions, medical examinations, and drug use.

    * It must cover and naturally integrate the dialogue snippets provided below.

5. Linguistic Features:

    * Concise, short, and direct.

    * Extremely **colloquial**. Sentence structures can be incomplete (e.g., omitted subjects, verbs, objects).

    * The logic in the patient's/family member's answers can be slightly confused or inconsistent.

    * The dialogue style must fit a natural, spoken Chinese context.

    * Use medical terms, but explain them when appropriate.

* The conversation should progress from general topics to specific ones.

* The conversation should be flexible, adapting based on the other person's responses.

Judgment Criteria

{common_prefix}

Required Dialogue Snippets

{input_text}

Expected Follow-up Report Results

{new_output_text}

The prompt for data Synthesis. Where {new_output_text} is a randomly generated result that serves as the ground truth for the synthetic dialogue.

Reference

1. Guo, B., Xing, W., Hu, C., et al. Clinical effectiveness of automated coronary CT-derived fractional flow reserve: A Chinese randomized controlled trial. *Radiology*. **313**:e233354. https://doi.org/10.1148/radiol.233354 (2024).
2. Fearon, W. F. et al. Fractional flow reserve-guided PCI as compared with coronary bypass surgery. *N Engl J Med*. **386,** 128-137. https://doi.org/10.1056/NEJMoa2112299 (2022).
3. Thygesen. K., et al. Third universal definition of myocardial infarction. *Eur Heart J.* **33,** 2551-2567. https://doi.org/10.1016/j.jacc.2012.08.001 (2012).
4. Ihdayhid, A. R., et al. Prognostic value and risk continuum of noninvasive fractional flow reserve derived from coronary CT angiography. *Radiology*. **292,** 343-351. https://doi.org/10.1148/radiol.2019182264 (2019).

Supplementary Tables

Supplementary Table 1 | Performances of Fu-LLM for adjudications of events with silver reference adjudications in each of the three centers.

	Raw agreement, % (95% CI)	Sensitivity, % (95% CI)	Specificity, % (95% CI)	Positive predictive value, % (95% CI)	Negative predictive value, % (95% CI)
**JL center**					
Whether the information came from the participant himself / herself	97.8 (96.3-99.4)	97.7 (95.2-100.0)	98.0 (95.6-100.0)	98.3 (96.0-100.0)	97.3 (94.6-99.4)
Whether the participant died	99.7 (99.1-100.0)	100.0 (100.0-100.0)	99.7 (99.0-100.0)	91.7 (72.7-100.0)	100.0 (100.0-100.0)
Whether the participant was hospitalized[#]	87.1 (83.2-90.6)	84.0 (75.4-92.2)	95.8 (92.7-98.3)	88.7 (80.5-95.7)	93.8 (90.4-96.8)
Whether the participant underwent surgery[#]	91.9 (88.7-94.8)	94.3 (87.5-100.0)	92.2 (88.1-95.8)	76.9 (66.7-86.7)	98.3 (96.2-100.0)
Whether the participant taken medication[#]	96.5 (94.5-98.1)	99.2 (98.0-100.0)	92.1 (82.6-100.0)	98.8 (97.4-100.0)	94.6 (86.1-100.0)
Total	94.7 (93.5-95.7)	96.3 (94.6-97.7)	96.6 (95.3-97.7)	94.8 (93.0-96.4)	97.6 (96.5-98.6)
**NFH center**					
Whether the information came from the participant himself / herself	98.3 (96.9-99.4)	99.2 (97.9-100.0)	96.6 (92.7-100.0)	98.3 (96.7-100.0)	98.3 (95.4-100.0)
Whether the participant died	100.0 (100.0-100.0)	100.0 (0.0-100.0)	100.0 (100.0-100.0)	100.0 (100.0-100.0)	100.0 (100.0-100.0)
Whether the participant was hospitalized[#]	83.8 (80.1-88.1)	94.0 (88.6-98.7)	92.3 (88.5-95.8)	83.0 (75.3-90.0)	97.5 (95.0-99.5)
Whether the participant underwent surgery[#]	92.3 (89.5-94.9)	95.4 (89.8-100.0)	91.8 (88.1-95.3)	78.5 (69.1-87.2)	98.5 (96.4-100.0)
Whether the participant taken medication[#]	96.9 (94.9-98.6)	100.0 (100.0-100.0)	85.7 (72.4-96.7)	98.4 (96.8-99.7)	100.0 (100.0-100.0)
Total	94.3 (93.1-95.4)	98.6 (97.6-99.4)	95.4 (94.0-96.7)	94.2 (92.6-95.9)	98.9 (98.2-99.6)
**JNU center**					
Whether the information came from the participant himself / herself	95.7 (93.8-97.8)	96.7 (94.1-99.0)	94.4 (90.7-98.0)	95.8 (92.8-98.4)	95.6 (92.2-98.7)
Whether the participant died	99.7 (99.2-100.0)	100.0 (100.0-100.0)	99.7 (99.2-100.0)	88.9 (63.6-100.0)	100.0 (100.0-100.0)

Whether the participant was hospitalized#	77.9 (73.8-82.0)	88.0 (78.6-96.4)	85.2 (79.4-90.3)	63.8 (52.7-75.4)	96.0 (92.8-98.7)
Whether the participant underwent surgery#	92.0 (89.2-94.8)	96.2 (90.5-100.0)	83.3 (76.7-89.2)	69.9 (59.2-80.0)	98.2 (95.4-100.0)
Whether the participant taken medication#	95.9 (93.6-97.8)	100.0 (100.0-100.0)	93.1 (86.4-98.4)	98.2 (96.4-99.6)	100.0 (100.0-100.0)
Total	92.3 (91.1-93.5)	97.5 (96.0-98.6)	93.1 (91.3-94.7)	90.2 (87.9-92.6)	98.2 (97.3-99.1)

#: For participants who were reported as dead during follow-up, for humanitarian reasons, the follow-up staff would not inquire about the information of hospitalization, surgery or medication, therefore these three events for the death cases will not be evaluated (22 recordings vignettes reported death events).

CI, confidence interval; *JL*, Jinling Hospital; *JNU*, The Affiliated Hospital of Jining Medical University; *NFH*, Nanjing First Hospital.

Supplementary Table 2 | Performances of Fu-LLM for adjudications of events with silver reference adjudications in each of the group assignment (CCTA+CT-FFR group vs CCTA group).

	Raw agreement, % (95% CI)	Sensitivity, % (95% CI)	Specificity, % (95% CI)	Positive predictive value, % (95% CI)	Negative predictive value, % (95% CI)
**Group of CCTA**					
Whether the information came from the participant himself / herself	96.7 (94.9-98.2)	97.6 (95.7-99.1)	95.4 (92.6-97.9)	96.6 (94.3-98.4)	96.8 (94.2-98.7)
Whether the participant died	99.8 (99.4-100.0)	100.0 (100.0-100.0)	99.8 (99.4-100.0)	93.3 (78.6-100.0)	100.0 (100.0-100.0)
Whether the participant was hospitalized#	82.2 (78.8-85.2)	87.0 (80.8-93.2)	90.9 (87.3-94.2)	79.7 (72.1-86.7)	94.5 (91.6-97.1)
Whether the participant underwent surgery#	92.6 (90.4-94.8)	93.3 (88.2-97.9)	90.5 (86.5-94.0)	77.1 (69.2-84.5)	97.5 (95.3-99.2)
Whether the participant taken medication#	95.6 (94.0-97.2)	99.7 (99.2-100.0)	91.0 (84.0-97.2)	98.2 (96.9-99.5)	98.6 (95.5-100.0)
Total	93.4 (92.4-94.4)	96.9 (95.7-98.0)	94.9 (93.7-96.1)	92.9 (91.2-94.5)	97.8 (97.0-98.6)
**Group of CCTA+CT-FFR**					
Whether the information came from the participant himself / herself	97.8 (96.4-98.9)	98.2 (96.7-99.4)	97.1 (94.5-99.1)	98.2 (96.7-99.7)	97.1 (94.7-99.0)
Whether the participant died	99.8 (99.4-100.0)	100.0 (100.0-100.0)	99.8 (99.4-100.0)	88.9 (62.5-100.0)	100.0 (100.0-100.0)
Whether the participant was hospitalized#	83.2 (80.0-86.1)	91.0 (84.5-96.2)	91.7 (88.7-94.5)	78.5 (70.2-86.0)	96.9 (94.8-98.6)
Whether the participant underwent surgery#	91.6 (89.1-93.9)	97.5 (93.8-100.0)	89.2 (85.3-92.8)	73.2 (64.1-81.6)	99.2 (97.9-100.0)
Whether the participant taken medication#	97.1 (95.6-98.5)	99.8 (99.3-100.0)	91.0 (83.1-97.3)	98.6 (97.5-99.5)	98.4 (94.4-100.0)
total	93.9 (93.0-94.8)	98.1 (97.2-98.9)	95.1 (94.0-96.2)	93.3 (91.8-94.9)	98.6 (98.0-99.2)
*P**	0.441	0.090	0.839	0.692	0.114

*, *p* indicates the differences of total raw agreement, sensitivity, specificity, positive predictive value and negative predictive value between the group of CCTA and the group of CCTA+CT-FFR.

#: For participants who were reported as dead during follow-up, for humanitarian reasons, the follow-up staff would not inquire about the information of hospitalization, surgery or medication, therefore these three events for the death cases will not be evaluated (22 recordings

vignettes reported death events).

Within-group differences of the overall agreement, sensitivity, specificity, NPV, and PPV between group of CCTA and group of CCTA+CT-FFR were assessed using χ^2 test. The statistical tests were 2-sided with significance set at $p < 0.05$.

CI, confidence interval; *JL*, Jinling Hospital; *JNU*, The Affiliated Hospital of Jining Medical University; *NFH*, Nanjing First Hospital..

Supplementary Table 3 | Performances of GPT-4 with Different Promts for Adjudications of Clinical Events.

	Raw agreement, % (95%CI)	Sensitivity, % (95%CI)	Specificity, % (95%CI)	Positive predictive value, % (95%CI)	Negative predictive value, % (95%CI)
**Performances of GPT-4 with zero_shot**					
Whether the information came from the participant himself / herself	94.8 (93.5-96.1)	98.2 (97.1-99.2)	89.9 (86.9-92.7)	93.4 (91.3-95.3)	97.2 (95.4-98.7)
Whether the participant died	99.9 (99.7-100.0)	100.0 (100.0-100.0)	99.9 (99.7-100.0)	95.7 (85.2-100.0)	100.0 (100.0-100.0)
Whether the participant was hospitalized[#]	71.6 (68.9-74.2)	83.2 (77.7-88.2)	90.8 (88.5-93.1)	76.9 (71.6-82.5)	93.6 (91.4-95.7)
Whether the participant underwent surgery[#]	74.8 (72.1-77.4)	86.6 (81.1-91.5)	97.2 (95.7-98.5)	90.8 (86.6-95.0)	95.7 (93.9-97.4)
Whether the participant taken medication[#]	96.2 (95.0-97.4)	98.8 (98.1-99.5)	87.6 (81.8-92.6)	97.8 (96.9-98.8)	92.7 (88.1-97.0)
Total	87.6 (86.7-88.4)	95.7 (94.9-96.6)	95.2 (94.4-96.0)	93.2 (92.1-94.3)	97.0 (96.3-97.6)
**Performances of GPT-4 with zero_shot_cot**					
Whether the information came from the participant himself / herself	94.3 (92.7-95.6)	97.4 (96.1-98.6)	89.6 (86.9-92.5)	93.2 (91.2-95.1)	96.0 (93.9-97.7)
Whether the participant died	99.5 (99.0-99.9)	100.0 (100.0-100.0)	99.5 (99.0-99.9)	81.5 (66.7-95.5)	100.0 (100.0-100.0)
Whether the participant was hospitalized[#]	60.4 (57.3-63.2)	88.0 (83.4-92.1)	62.7 (58.5-66.7)	46.5 (41.2-51.5)	93.4 (90.8-95.8)
Whether the participant underwent surgery[#]	79.9 (77.3-82.3)	91.2 (86.9-95.5)	90.8 (88.3-93.1)	76.1 (70.4-81.9)	97.0 (95.3-98.4)
Whether the participant taken medication[#]	95.6 (94.3-96.8)	99.0 (98.3-99.6)	82.8 (76.3-88.5)	97.0 (95.8-98.1)	93.8 (89.4-97.4)
Total	86.0 (85.1-87.1)	96.5 (95.6-97.3)	87.6 (86.5-88.8)	84.3 (82.7-85.7)	97.4 (96.7-97.9)
**Performances of GPT-4 with one_shot**					
Whether the information came from the participant himself / herself	95.3 (94.1-96.7)	96.5 (94.9-97.9)	93.6 (91.3-96.0)	95.7 (94.1-97.2)	94.8 (92.6-96.7)
Whether the participant died	99.8 (99.5-100.0)	100.0 (100.0-100.0)	99.8 (99.5-100.0)	91.7 (78.6-100.0)	100.0 (100.0-100.0)
Whether the participant was hospitalized[#]	60.4 (57.3-63.2)	83.7 (78.4-88.7)	87.8 85.2-90.5)	71.6 (65.6-76.8)	93.6 (91.5-95.6)
Whether the participant underwent surgery[#]	73.6 (70.9-76.3)	86.6 (81.0-91.6)	95.9 (94.0-97.5)	87.1 (81.9-91.8)	95.7 (94.0-97.3)

Whether the participant taken medication[#]	94.7 (93.5-96.0)	96.6 (95.3-97.8)	93.1 (88.4-97.0)	98.8 (98.0-99.4)	82.8 (77.0-87.9)
Total	86.8 (85.9-87.7)	94.2 (93.2-95.2)	95.2 (94.3-95.9)	93.1 (91.9-94.2)	96.0 (95.3-96.7)

[#] : For participants who were reported as dead during follow-up, for humanitarian reasons, the follow-up staff would not inquire about the information of hospitalization, surgery or medication, therefore these three events of the death cases will not be evaluated (22 recordings vignettes reported death events).

CI, confidence interval; *GPT*, Generative pretrained transformer..

Supplementary Table 4 | Performances of GPT-4 with zero_shot at different timepoints for Adjudications of Clinical Events from data of JL center.

	Raw agreement, % (95%CI)	Sensitivity, % (95%CI)	Specificity, % (95%CI)	Positive predictive value, % (95%CI)	Negative predictive value, % (95%CI)
**First timepoints (September 2023)**					
Whether the information came from the participant himself / herself	96.6 (94.7-98.4)	98.8 (97.1-100.0)	93.9 (89.8-97.3)	95.0 (91.8-97.8)	98.6 (96.5-100.0)
Whether the participant died	98.8 (97.5-99.7)	100.0 (100.0-100.0)	98.7 (97.4-99.7)	73.3 (50.0-94.1)	100.0 (100.0-100.0)
Whether the participant was hospitalized#	65.2 (60.0-70.7)	80.0 (69.8-88.6)	70.9 (64.2-77.1)	52.2 (43.9-61.8)	89.9 (84.9-94.6)
Whether the participant underwent surgery#	82.9 (78.7-87.1)	92.5 (84.7-98.3)	96.9 (94.3-99.0)	89.1 (80.0-96.4)	97.9 (95.7-99.5)
Whether the participant taken medication#	93.2 (90.3-96.1)	99.6 (98.8-100.0)	81.6 (68.7-93.0)	97.3 (95.3-99.2)	96.9 (89.5-100.0)
Total	87.5 (85.8-89.1)	96.1 (94.4-97.6)	90.8 (88.9-92.6)	87.0 (84.4-89.5)	97.3 (96.1-98.3)
**Second timepoints (January 2024)**					
Whether the information came from the participant himself / herself	98.1 (96.6-99.4)	99.4 (98.1-100.0)	96.6 (89.8-97.3)	97.2 (94.5-99.4)	99.3 (97.8-100.0)
Whether the participant died	96.0 (93.8-98.1)	100.0 (100.0-100.0)	95.8 (93.5-97.7)	45.8 (25.0-65.0)	100.0 (100.0-100.0)
Whether the participant was hospitalized#	44.8 (39.7-51.0)	73.3 (61.8-83.3)	38.6 (32.0-45.4)	32.2 (25.6-39.3)	78.5 (69.7-85.7)
Whether the participant underwent surgery#	81.0 (76.8-85.2)	90.6 (82.1-97.9)	84.8 (79.7-89.8)	62.3 (51.4-72.8)	97.0 (94.0-99.4)
Whether the participant taken medication#	96.1 (93.5-98.1)	98.4 (96.8-99.6)	86.8 (74.2-97.1)	98.0 (96.1-99.6)	89.2 (78.6-97.7)
Total	83.4 (81.6-85.2)	94.7 (92.7-96.7)	80.8 (78.1-83.4)	76.1 (72.9-79.3)	95.9 (94.5-97.4)
**Third timepoints (April 2025)**					
Whether the information came from the participant himself / herself	94.4 (91.9-96.6)	90.8 (86.3-94.6)	98.7 (96.6-100.0)	98.7 (96.8-100.0)	90.1 (85.6-94.4)
Whether the participant died	99.7 (99.1-100.0)	100.0 (100.0-100.0)	99.7 (99.0-100.0)	91.7 (72.7-100.0)	100.0 (100.0-100.0)

Whether the participant was hospitalized[#]	75.2 (70.3-79.7)	69.3 (58.6-79.2)	95.8 (92.7-98.4)	86.7 (77.6-94.5)	88.7 (84.2-93.0)
Whether the participant underwent surgery[#]	75.5 (70.6-79.7)	92.5 (84.9-98.2)	96.9 (93.9-99.0)	89.1 (81.0-96.4)	97.9 (95.6-99.5)
Whether the participant taken medication[#]	91.0 (87.7-93.9)	98.4 (96.7-99.6)	86.8 (75.7-97.0)	98.0 (96.2-99.6)	89.2 (78.6-97.4)
Total	87.3 (85.6-88.9)	91.7 (89.4-94.1)	97.5 (96.4-98.4)	95.9 (94.0-97.5)	94.8 (93.3-96.2)

[#]: For participants who were reported as dead during follow-up, for humanitarian reasons, the follow-up staff would not inquire about the information of hospitalization, surgery or medication, therefore these three events for the death cases will not be evaluated (22 recordings vignettes reported death events).

CI, confidence interval.

Supplementary Table 5 | Performances of Five Popular Public LLMs for Adjudications of Clinical Events.

	Raw agreement, % (95%CI)	Sensitivity, % (95%CI)	Specificity, % (95%CI)	Positive predictive value, % (95%CI)	Negative predictive value, % (95%CI)
**Performances of DeepSeek-v3 (2024_12_26)**					
Whether the information came from the participant himself / herself	95.5 (94.3-96.7)	96.3 (94.6-97.7)	94.3 (92.2-96.4)	96.2 (94.7-97.6)	94.6 (92.5-96.5)
Whether the participant died	99.6 (99.2-99.9)	100.0 (100.0-100.0)	99.6 (99.2-99.9)	84.6 (70.0-100.0)	100.0 (100.0-100.0)
Whether the participant was hospitalized#	32.6 (29.9-35.5)	75.0 (68.3-80.8)	16.8 (14.0-20.2)	24.9 (21.6-28.4)	64.6 (56.5-72.4)
Whether the participant underwent surgery#	89.1 (87.3-90.9)	94.7 (91.1-97.8)	86.3 (83.2-88.9)	68.9 (63.0-74.8)	98.7 (96.7-99.2)
Whether the participant taken medication#	95.2 (93.9-96.4)	97.7 (96.6-98.7)	92.4 (87.8-96.7)	98.7 (97.9-99.4)	87.6 (82.0-92.6)
Total	82.5 (81.5-83.5)	94.4 (93.4-95.4)	78.3 (76.8-79.8)	75.0 (73.2-76.8)	95.3 (94.4-96.2)
**Performances of GPT-3.5-turbo (2025_01_25)**					
Whether the information came from the participant himself / herself	88.5 (86.6-90.4)	93.6 (91.6-95.4)	81.1 (77.2-84.5)	87.9 (85.4-90.4)	89.6 (92.5-96.5)
Whether the participant died	99.3 (98.9-99.8)	100.0 (100.0-100.0)	99.3 (98.8-99.7)	75.9 (57.1-90.5)	100.0 (100.0-100.0)
Whether the participant was hospitalized#	65.0 (61.9-68.0)	69.2 (62.6-75.1)	89.8 (87.3-92.1)	71.3 (64.8-77.0)	88.8 (86.3-91.4)
Whether the participant underwent surgery#	67.2 (64.3-70.0)	91.8 (87.2-95.8)	95.1 (93.1-96.8)	85.8 (80.2-90.7)	97.3 (95.8-98.6)
Whether the participant taken medication#	91.8 (90.1-93.5)	94.8 (93.2-96.3)	93.1 (88.7-96.8)	98.7 (98.0-99.5)	75.8 (69.2-82.6)
Total	82.5 (81.4-83.5)	91.3 (90.1-92.6)	93.3 (92.3-94.2)	90.3 (89.0-91.6)	94.0 (93.0-94.9)
**Performances of GPT-4o (2024_11_20)**					
Whether the information came from the participant himself / herself	96.1 (94.9-97.2)	96.8 (95.4-98.0)	95.1 (92.9-97.0)	96.6 (95.2-97.9)	95.3 (93.2-97.0)
Whether the participant died	99.9 (99.7-100.0)	100.0 (100.0-100.0)	99.9 (99.6-100.0)	95.7 (85.0-100.0)	100.0 (100.0-100.0)
Whether the participant was hospitalized#	63.3 (60.3-66.3)	52.4 (45.3-58.9)	88.5 (85.9-91.1)	62.6 (55.9-69.9)	83.5 (80.1-86.4)
Whether the participant underwent surgery#	73.7 (71.2-76.5)	90.1 (85.6-94.4)	96.4 (94.9-97.9)	89.0 (84.1-93.6)	96.8 (95.3-98.2)

Whether the participant taken medication#	95.1 (93.8-96.4)	97.3 (96.2-98.4)	93.8 (89.8-97.7)	98.9 (98.1-99.5)	86.1 (80.4-91.0)
Total	85.7 (84.6-86.6)	91.5 (90.1-92.7)	95.7 (95.0-96.5)	93.6 (92.5-94.7)	94.2 (93.3-95.0)
**Performances of claude 3.5-sonnet (2024_10_22)**					
Whether the information came from the participant himself / herself	95.5 (94.3-96.7)	98.6 (97.5-99.4)	91.0 (88.3-93.5)	94.2 (92.3-96.0)	97.7 (96.1-99.0)
Whether the participant died	99.9 (99.7-100.0)	100.0 (100.0-100.0)	99.9 (99.7-100.0)	95.7 (86.2-100.0)	100.0 (100.0-100.0)
Whether the participant was hospitalized#	68.5 (65.4-71.2)	78.9 (73.8-83.9)	92.6 (90.3-94.7)	79.6 (73.7-85.4)	92.3 (89.9-94.2)
Whether the participant underwent surgery#	71.0 (68.3-73.7)	93.0 (88.6-96.8)	97.7 (96.4-98.9)	93.0 (88.9-96.3)	97.7 (96.4-98.9)
Whether the participant taken medication#	95.1 (93.8-96.4)	98.2 (97.2-98.9)	91.0 (86.0-96.0)	98.4 (97.4-99.3)	89.8 (84.8-94.8)
Total	86.1 (85.1-87.0)	95.7 (94.7-96.5)	96.1 (95.3-96.8)	94.4 (93.3-95.4)	97.0 (96.3-97.6)
**Performances of gemini-2.0-pro (2025_02_05)**					
Whether the information came from the participant himself / herself	95.8 (94.5-96.9)	97.9 (96.7-98.9)	92.7 (90.2-95.1)	95.2 (93.5-96.9)	96.8 (95.0-98.3)
Whether the participant died	99.8 (99.5-100.0)	100.0 (100.0-100.0)	99.8 (99.5-100.0)	91.7 (80.0-100.0)	100.0 (100.0-100.0)
Whether the participant was hospitalized#	65.1 (62.2-68.1)	58.7 (52.3-65.1)	96.3 (94.7-97.7)	85.3 (79.5-91.0)	86.4 (83.8-88.9)
Whether the participant underwent surgery#	65.4 (62.6-68.6)	95.9 (92.7-98.7)	95.1 (93.2-96.9)	86.3 (81.2-91.2)	98.6 (97.5-99.6)
Whether the participant taken medication#	93.4 (91.8-94.8)	97.6 (96.5-98.7)	93.8 (89.6-97.5)	98.9 (98.1-99.5)	87.2 (81.8-92.1)
Total	84.0 (83.0-85.0)	93.2 (92.0-94.2)	96.7 (96.0-97.4)	95.1 (94.1-96.0)	95.4 (94.6-96.2)

# : For participants who were reported as dead during follow-up, for humanitarian reasons, the follow-up staff would not inquire about the information of hospitalization, surgery or medication, therefore these three events of the death cases will not be evaluated (22 recordings vignettes reported death events).

CI, confidence interval; *GPT*, Generative pretrained transformer.

Supplementary Table 6 | Performances of SVMs for Adjudications of Events with Silver Reference Adjudications.

	Raw agreement, % (95%CI)	Sensitivity, % (95%CI)	Specificity, % (95%CI)	Positive predictive value, % (95%CI)	Negative predictive value, % (95%CI)
**Performances of svm_w2v**					
Whether the information came from the participant himself / herself	84.3 (82.0-86.4)	95.8 (94.1-97.4)	67.5 (63.1-72.2)	81.2 (78.4-83.8)	91.7 (88.5-94.7)
Whether the participant died	97.9 (97.0-98.8)	0.0 (0.0-0.0)	100.0 (100.0-100.0)	0.0 (0.0-0.0)	97.9 (97.0-98.7)
Whether the participant was hospitalized#	67.1 (64.3-69.9)	46.2 (39.1-53.5)	96.1 (94.4-97.7)	81.4 (74.3-88.4)	82.9 (80.0-85.8)
Whether the participant underwent surgery#	70.1 (67.6-72.9)	34.5 (28.0-41.7)	84.9 (81.8-87.7)	42.5 (34.1-50.0)	80.1 (76.9-83.2)
Whether the participant taken medication#	86.7 (84.6-88.8)	99.0 (98.3-99.6)	39.3 (31.6-47.1)	90.3 (88.3-92.1)	87.7 (78.9-95.2)
Total	81.3 (80.3-82.3)	84.9 (83.2-86.6)	87.8 (86.4-89.0)	82.7 (81.1-84.2)	89.4 (88.2-90.6)
**Performances of svm_w2v_wo_aug**					
Whether the information came from the participant himself / herself	60.1 (57.3-63.0)	99.8 (99.4-100.0)	1.9 (0.7-3.3)	59.9 (56.9-62.9)	88.9 (60.0-100.0)
Whether the participant died	97.9 (96.9-98.8)	0.0 (0.0-0.0)	100.0 (100.0-100.0)	0.0 (0.0-0.0)	97.9 (97.0-98.7)
Whether the participant was hospitalized#	56.0 (52.9-59.0)	0.5 (0.0-1.6)	99.1 (98.2-99.8)	16.7 (0.0-50.0)	73.1 (69.8-76.1)
Whether the participant underwent surgery#	59.4 (56.3-62.4)	0.0 (0.0-0.0)	96.1 (94.3-97.7)	0.0 (0.0-0.0)	74.9 (71.8-77.9)
Whether the participant taken medication#	80.8 (78.3-83.2)	100.0 (100.0-100.0)	0.0 (0.0-0.0)	85.1 (82.7-87.3)	0.0 (0.0-0.0)
Total	70.9 (69.6-72.2)	78.3 (76.5-80.2)	78.2 (76.6-79.6)	71.2 (69.2-73.1)	84.0 (82.5-85.4)
**Performances of svm_tfidf**					
Whether the information came from the participant himself / herself	84.0 (82.1-86.2)	93.3 (91.2-95.1)	70.5 (66.4-74.8)	82.3 (79.4-84.9)	87.7 (84.1-91.0)
Whether the participant died	98.7 (97.9-99.3)	40.9 (20.0-61.5)	99.9 (99.7-100.0)	90.0 (66.7-100.0)	98.8 (98.1-99.4)
Whether the participant was hospitalized#	66.4 (63.7-69.3)	38.5 (31.3-45.1)	95.1 (93.2-96.7)	74.1 (65.5-81.7)	80.8 (78.0-83.4)

Whether the participant underwent surgery#	73.2 (70.7-76.0)	28.7 (21.9-35.8)	87.2 (84.5-89.9)	41.9 (33.0-50.9)	79.2 (75.6-82.0)
Whether the participant taken medication#	82.3 (79.8-84.7)	99.9 (99.6-100.0)	9.0 (4.8-13.9)	86.2 (83.8-88.3)	92.9 (76.9-100.0)
Total	81.0 (79.9-82.0)	83.5 (81.7-85.3)	86.8 (85.7-88.2)	81.4 (79.5-83.0)	88.4 (87.2-89.6)
**Performances of svm_tfidf_wo_aug**					
Whether the information came from the participant himself / herself	78.4 (75.8-80.8)	96.1 (94.6-97.5)	52.4 (47.7-56.7)	74.8 (71.6-77.9)	90.2 (86.4-93.8)
Whether the participant died	98.1 (97.2-98.9)	9.1 (0.0-23.1)	100.0 (100.0-100.0)	100.0 (0.0-100.0)	98.1 (97.2-98.9)
Whether the participant was hospitalized#	59.8 (56.8-62.6)	23.1 (17.4-28.6)	97.0 (95.4-98.3)	73.9 (62.7-84.1)	77.4 (74.3-80.7)
Whether the participant underwent surgery#	69.9 (67.1-72.9)	18.7 (13.2-24.6)	91.2 (88.7-93.3)	40.5 (30.0-51.0)	77.7 (74.4-80.9)
Whether the participant taken medication#	81.0 (78.6-83.3)	100.0 (100.0-100.0)	1.4 (0.0-3.5)	85.3 (83.0-87.4)	100.0 (0.0-100.0)
Total	77.5 (76.4-78.7)	81.5 (79.6-83.2)	84.8 (83.5-86.1)	78.7 (76.8-80.6)	86.9 (85.7-88.2)

#: For participants who were reported as dead during follow-up, for humanitarian reasons, the follow-up staff would not inquire about the information of hospitalization, surgery or medication, therefore these three events of the death cases will not be evaluated (22 recordings vignettes reported death events).

CI, confidence interval; SVM, Support Vector Machine.

Supplementary Table 7 | Performances of Fu-LLM and human staffs for Adjudications of Clinical Events from data of JNU center.

	Raw agreement, % (95%CI)	Sensitivity, % (95%CI)	Specificity, % (95%CI)	Positive predictive value, % (95%CI)	Negative predictive value, % (95%CI)
**Fu-LLM**					
Whether the information came from the participant himself / herself	95.7 (93.8-97.8)	96.7 (94.1-99.0)	94.4 (90.7-98.0)	95.8 (92.8-98.4)	95.6 (92.2-98.7)
Whether the participant died	99.7 (99.2-100.0)	100.0 (100.0-100.0)	99.7 (99.2-100.0)	88.9 (63.6-100.0)	100.0 (100.0-100.0)
Whether the participant was hospitalized#	77.9 (73.8-82.0)	88.0 (78.6-96.4)	85.2 (79.4-90.3)	63.8 (52.7-75.4)	96.0 (92.8-98.7)
Whether the participant underwent surgery#	92.0 (89.2-94.8)	96.2 (90.5-100.0)	83.3 (76.7-89.2)	69.9 (59.2-80.0)	98.2 (95.4-100.0)
Whether the participant taken medication#	95.9 (93.6-97.8)	100.0 (100.0-100.0)	93.1 (86.4-98.4)	98.2 (96.4-99.6)	100.0 (100.0-100.0)
Total	92.3 (91.1-93.5)	97.5 (96.0-98.6)	93.1 (91.3-94.7)	90.2 (87.9-92.6)	98.2 (97.3-99.1)
**Human staffs**					
Whether the information came from the participant himself / herself	89.5 (88.0-91.1)	90.5 (88.4-92.5)	88.3 (85.8-90.7)	91.0 (89.1-92.8)	87.6 (85.0-90.2)
Whether the participant died	99.7 (99.5-99.9)	100.0 (100.0-100.0)	99.7 (99.4-99.9)	88.9 (78.0-97.5)	100.0 (100.0-100.0)
Whether the participant was hospitalized#	65.2 (62.8-67.8)	56.5 (49.4-63.4)	67.2 (63.7-70.8)	33.7 (28.8-39.0)	83.9 (80.8-87.1)
Whether the participant underwent surgery#	72.9 (70.7-75.3)	55.7 (48.9-62.4)	77.8 (74.1-81.5)	50.2 (44.0-56.9)	81.4 (78.0-84.9)
Whether the participant taken medication#	89.2 (87.4-90.8)	92.5 (91.0-94.1)	83.3 (78.9-87.1)	95.4 (94.1-96.6)	75.0 (70.3-79.5)
Total	83.4 (82.6-84.3)	85.5 (84.1-86.8)	87.0 (85.8-88.1)	81.2 (79.7-82.6)	90.1 (89.1-91.1)
**Staff 1**					
Whether the information came from the participant himself / herself	85.7 (82.2-88.9)	90.5 (86.4-94.0)	79.4 (73.2-85.8)	85.2 (80.1-89.5)	86.4 (80.1-91.5)
Whether the participant died	99.5 (98.6-100.0)	100.0 (100.0-100.0)	99.5 (98.6-100.0)	80.0 (50.0-100.0)	100.0 (100.0-100.0)
Whether the participant was hospitalized#	47.2 (42.8-52.2)	86.0 (76.5-95.3)	40.2 (33.1-47.6)	29.9 (23.1-37.7)	90.7 (83.3-96.4)
Whether the participant underwent surgery#	82.3 (78.2-86.2)	83.0 (72.7-92.0)	72.0 (64.3-80.2)	54.3 (43.1-65.4)	91.4 (85.2-96.3)

Whether the participant taken medication#	85.6 (82.0-89.0)	86.5 (82.1-90.4)	86.1 (78.4-93.5)	95.9 (93.4-98.0)	63.3 (53.3-72.6)
Total	80.2 (78.3-81.9)	87.8 (85.0-90.3)	79.6 (76.9-82.1)	73.8 (70.5-77.1)	90.8 (88.7-92.7)
**Staff 2**					
Whether the information came from the participant himself / herself	94.3 (91.9-96.5)	96.2 (93.4-98.6)	91.9 (87.4-96.1)	94.0 (90.5-96.9)	94.8 (91.1-98.0)
Whether the participant died	99.7 (99.2-100.0)	100.0 (100.0-100.0)	99.7 (98.9-100.0)	88.9 (63.6-100.0)	100.0 (100.0-100.0)
Whether the participant was hospitalized#	73.8 (69.3-78.5)	38.0 (24.4-51.2)	80.5 (74.3-85.8)	36.5 (23.6-49.1)	81.4 (74.7-87.3)
Whether the participant underwent surgery#	80.1 (76.2-84.0)	26.4 (15.3-38.9)	78.8 (72.2-85.1)	33.3 (18.9-47.6)	72.7 (65.2-79.5)
Whether the participant taken medication#	91.2 (88.4-93.9)	94.8 (91.9-97.3)	77.8 (67.6-87.0)	94.1 (90.7-96.7)	80.0 (70.6-89.2)
Total	87.9 (86.5-89.3)	84.4 (81.2-87.1)	89.8 (87.7-91.6)	84.5 (81.6-87.4)	89.7 (87.6-91.6)
**Staff 3**					
Whether the information came from the participant himself / herself	91.6 (88.6-94.3)	92.4 (88.6-95.9)	90.6 (85.7-95.3)	92.8 (80.1-89.5)	90.1 (85.2-94.5)
Whether the participant died	99.7 (99.2-100.0)	100.0 (100.0-100.0)	99.7 (99.2-100.0)	88.9 (63.6-100.0)	100.0 (100.0-100.0)
Whether the participant was hospitalized#	73.2 (69.1-77.9)	50.0 (36.4-65.0)	71.0 (64.4-77.1)	33.8 (23.3-45.7)	82.8 (76.9-88.8)
Whether the participant underwent surgery#	81.5 (77.3-85.4)	52.8 (39.6-66.1)	72.7 (64.7-80.3)	43.8 (30.8-55.6)	79.3 (71.6-87.4)
Whether the participant taken medication#	89.2 (86.2-92.3)	95.1 (92.4-97.6)	76.4 (66.2-85.2)	93.7 (90.4-96.3)	80.9 (70.8-89.5)
Total	87.1 (85.5-88.7)	86.6 (83.6-89.0)	86.8 (84.6-89.0)	81.2 (78.0-84.3)	90.8 (88.6-92.7)
**Staff 4**					
Whether the information came from the participant himself / herself	86.5 (83.0-89.7)	82.9 (77.4-87.9)	91.3 (86.9-95.2)	92.6 (88.8-96.1)	80.2 (74.2-86.0)
Whether the participant died	100.0 (100.0-100.0)	100.0 (100.0-100.0)	100.0 (100.0-100.0)	100.0 (100.0-100.0)	100.0 (100.0-100.0)
Whether the participant was hospitalized#	66.6 (61.9-71.3)	52.0 (38.0-65.9)	76.9 (70.3-82.9)	40.0 (28.8-51.9)	84.4 (79.0-89.6)
Whether the participant underwent surgery#	47.8 (42.5-53.0)	60.4 (46.8-72.4)	87.9 (82.1-93.0)	66.7 (53.8-79.5)	84.7 (78.6-90.5)

Whether the participant taken medication#	90.6 (87.6-93.6)	93.6 (90.6-96.2)	93.1 (86.9-98.5)	98.0 (96.2-99.6)	79.8 (70.6-87.9)
Total	78.4 (76.6-80.3)	83.3 (80.2-86.4)	91.7 (90.0-93.4)	86.9 (84.3-89.4)	89.3 (87.4-91.2)

#: For participants who were reported as dead during follow-up, for humanitarian reasons, the follow-up staff would not inquire about the information of hospitalization, surgery or medication, therefore these three events for the death cases will not be evaluated (22 recordings vignettes reported death events).

CI, confidence interval.
