# Supplementary material for: A large language model for clinical outcome adjudication from telephone follow-up interviews: a secondary analysis of a multicenter randomized clinical trial
Source: Nat Commun. 2025 Dec 1;17:211. doi: 10.1038/s41467-025-66910-6 (PMC12779956; doi:10.1038/s41467-025-66910-6)
Supplement: Supplementary file 2 — Reporting Summary [file 41467_2025_66910_MOESM2_ESM.pdf]

nature portfolio

Corresponding author(s): Long Jiang Zhang

Last updated by author(s): Oct 9, 2025

Reporting Summary

Nature Portfolio wishes to improve the reproducibility of the work that we publish. This form provides structure for consistency and transparency in reporting. For further information on Nature Portfolio policies, see our Editorial Policies and the Editorial Policy Checklist.

Statistics

For all statistical analyses, confirm that the following items are present in the figure legend, table legend, main text, or Methods section.

n/a	Confirmed	
☐	☒	The exact sample size (*n*) for each experimental group/condition, given as a discrete number and unit of measurement
☐	☒	A statement on whether measurements were taken from distinct samples or whether the same sample was measured repeatedly
☐	☒	The statistical test(s) used AND whether they are one- or two-sided<br>*Only common tests should be described solely by name; describe more complex techniques in the Methods section.*
☐	☒	A description of all covariates tested
☐	☒	A description of any assumptions or corrections, such as tests of normality and adjustment for multiple comparisons
☐	☒	A full description of the statistical parameters including central tendency (e.g. means) or other basic estimates (e.g. regression coefficient) AND variation (e.g. standard deviation) or associated estimates of uncertainty (e.g. confidence intervals)
☐	☒	For null hypothesis testing, the test statistic (e.g. *F*, *t*, *r*) with confidence intervals, effect sizes, degrees of freedom and *P* value noted<br>*Give P values as exact values whenever suitable.*
☒	☐	For Bayesian analysis, information on the choice of priors and Markov chain Monte Carlo settings
☒	☐	For hierarchical and complex designs, identification of the appropriate level for tests and full reporting of outcomes
☐	☒	Estimates of effect sizes (e.g. Cohen's *d*, Pearson's *r*), indicating how they were calculated

Our web collection on statistics for biologists contains articles on many of the points above.

Software and code

Policy information about availability of computer code

Data collection	The code of Fu-LLM is deposited in Zenodo (https://doi.org/10.5281/zenodo.17221355), and is also available at GitHub (https://github.com/OmniMedAI/FuLLM).<br>Gpt-4 (2023.09, 2024.01, 2025.04), GPT-3.5-turbo (2025_01_25), GPT-4o (2024_11_20), DeepSeek-v3 (2024_12_26), claude 3.5-sonnet (2024_10_22) and gemini-2.0-pro (2025_02_05) are public State-of-the-Art LLM.
Data analysis	Statistical analyses were performed using SPSS Statistics (version 22.0.0; IBM) and Python (version 3.7.6).

For manuscripts utilizing custom algorithms or software that are central to the research but not yet described in published literature, software must be made available to editors and reviewers. We strongly encourage code deposition in a community repository (e.g. GitHub). See the Nature Portfolio guidelines for submitting code & software for further information.

Data

Policy information about availability of data

All manuscripts must include a data availability statement. This statement should provide the following information, where applicable:

- Accession codes, unique identifiers, or web links for publicly available datasets
- A description of any restrictions on data availability
- For clinical datasets or third party data, please ensure that the statement adheres to our policy

Prof. L.J.Z. managed the data and was responsible for the integrity of the data. The raw data has been depiosited in Figshare (Figshare DOI: https://doi.org/10.6084/m9.figshare.25498102). The data generated in this study are provided in the Source Data file. Source data are provided with this paper.

Research involving human participants, their data, or biological material

Policy information about studies with human participants or human data. See also policy information about sex, gender (identity/presentation), and sexual orientation and race, ethnicity and racism.

Reporting on sex and gender	We collected the data of a multicenter, prospective randomized clinical trial (China CT-FFR Study 3) and did not consider sex in study design.<br>Sex was recorded based on assigned at birth.<br>In the retrospective data, a total of 1,191 vignettes of telephone interviews of follow-up were collected from the three hospitals and 1,046 were included for final analysis, and there were 619 male and 403 female individuals included in this study.<br>Sex has been used as a covariate to evaluate Fu-LLM's performance. Findings were applied to both sex.
Reporting on race, ethnicity, or other socially relevant groupings	Race, ethnicity, or other socially relevant groupings were not collected in this study and were unrelated to Fu-LLM's development or performance.
Population characteristics	A full description of population characteristics in the study cohort was given in Table 1.<br>1,022 participants (mean [standard deviation, SD] age, 63.1 [11.0] years; 403 [39.4%] female, 619 [60.6%] male) were included in this study, who were assigned to CCTA alone (n=498, 48.7%) or CCTA+CT-FFR (n=524, 51.3%) groups.
Recruitment	In this secondary analysis, three centers (Jinling Hospital [JL center], Nanjing First Hospital [NFH center], and The Affiliated Hospital of Jining Medical University [JNU center]) from a multicenter, prospective randomized clinical trial (China CT-FFR Study 3) with sound recordings of 1-year follow-up telephone interview saved were included. The inclusion criterion of the secondary study was participants who had completed 1-year telephone interview follow-up with corresponding sound recordings saved in the clinical trial follow-up system (n=1,191). The exclusion criteria were: (1) Recordings with participant unwilling to respond (n=27); (2) Failed recordings (n=34); (3) Poor recording quality, such as strong background noise affecting judgement (n=62); (4) Recordings that were unrecognizable due to heavy local dialect and unclear pronunciation (n=22).
Ethics oversight	This secondary analysis aimed to develop a domain-specific follow-up LLM (Fu-LLM) and investigate the feasibility of the model to automatically adjudicate participants' clinical outcomes from telephone interview, which had also been approved by ethics committee of Jinling Hospital (ID: 2024DZKY-022-01) and had been registered (ChiCTR.org.cn Identifier: ChiCTR2400080585).

Note that full information on the approval of the study protocol must also be provided in the manuscript.

Field-specific reporting

Please select the one below that is the best fit for your research. If you are not sure, read the appropriate sections before making your selection.

☒ Life sciences ☐ Behavioural & social sciences ☐ Ecological, evolutionary & environmental sciences

For a reference copy of the document with all sections, see nature.com/documents/nr-reporting-summary-flat.pdf

Life sciences study design

All studies must disclose on these points even when the disclosure is negative.

Sample size	No formal a priori sample size calculation was performed. This secondary analysis aimed to develop a domain-specific follow-up LLM (Fu-LLM) and investigate the feasibility of the model to automatically adjudicate participants' clinical outcomes from telephone interview. The sample consisted of all available and eligible telephone interview recordings (n=1,046) from three participating centers in the parent trial (China CT-FFR Study 3) that met quality standards. To enhance robustness despite the limited original dataset, extensive data augmentation was employed—generating 19,162 training samples—and model evaluation was conducted using five-fold cross-validation. This approach maximizes the utility of available data and provides reliable performance estimates, which is standard practice in machine learning studies where fixed sample sizes are constrained by real-world data availability. The high accuracy achieved (e.g., 93.7% raw agreement) supports the sufficiency of the sample size for model development and evaluation.

Data exclusions	The exclusion criteria were: (1) Recordings with participant unwilling to respond (n=27); (2) Failed recordings (n=34); (3) Poor recording quality, such as strong background noise affecting judgement (n=62); (4) Recordings that were unrecognizable due to heavy local dialect and unclear pronunciation (n=22).
Replication	All attempts at replication were successful. The performance of Fu-LLM was consistent across the three centers and different arms.
Randomization	Randomization is not relevant to the study, for that Fu-LLM processed all cases without grouping.
Blinding	For this secondary analysis, blinding was not feasible nor directly relevant to the primary objective. The study focused on developing and validating an AI model (Fu-LLM) to automate the extraction of clinical outcomes from dialogue text, rather than comparing outcomes between the original trial's randomized groups (CCTA vs. CCTA+CT-FFR). Model performance was evaluated against silver reference standards derived from human-adjudicated labels, not based on treatment group assignment. Thus, knowledge of group allocation did not introduce bias in model training or outcome adjudication tasks.

Reporting for specific materials, systems and methods

We require information from authors about some types of materials, experimental systems and methods used in many studies. Here, indicate whether each material, system or method listed is relevant to your study. If you are not sure if a list item applies to your research, read the appropriate section before selecting a response.

Materials & experimental systems

n/a	Involved in the study
☒	☐ Antibodies
☒	☐ Eukaryotic cell lines
☒	☐ Palaeontology and archaeology
☒	☐ Animals and other organisms
☒	☐ Clinical data
☒	☐ Dual use research of concern
☒	☐ Plants

Methods

n/a	Involved in the study
☒	☐ ChIP-seq
☒	☐ Flow cytometry
☒	☐ MRI-based neuroimaging

Plants

Seed stocks	*Report on the source of all seed stocks or other plant material used. If applicable, state the seed stock centre and catalogue number. If plant specimens were collected from the field, describe the collection location, date and sampling procedures.*
Novel plant genotypes	*Describe the methods by which all novel plant genotypes were produced. This includes those generated by transgenic approaches, gene editing, chemical/radiation-based mutagenesis and hybridization. For transgenic lines, describe the transformation method, the number of independent lines analyzed and the generation upon which experiments were performed. For gene-edited lines, describe the editor used, the endogenous sequence targeted for editing, the targeting guide RNA sequence (if applicable) and how the editor was applied.*
Authentication	*Describe any authentication procedures for each seed stock used or novel genotype generated. Describe any experiments used to assess the effect of a mutation and, where applicable, how potential secondary effects (e.g. second site T-DNA insertions, mosiacism, off-target gene editing) were examined.*
